# Effective practices during emergency school lockdowns: shared experiences of four Australian schools

**DOI:** 10.1007/s13384-022-00588-3

**Published:** 2022-11-12

**Authors:** M. Kearney, S. Schuck, J. Fergusson, R. Perry

**Affiliations:** 1 Faculty of Arts and Social Sciences, University of Technology Sydney (UTS), Ultimo, Australia; 2 The Association of Independent Schools of NSW, Sydney, Australia

**Keywords:** Emergency remote teaching, School education, Pedagogical practices, Wellbeing, Inclusion

## Abstract

This study investigates common features of a set of diverse schools’ responses to the initial school lockdown period during the pandemic in 2020, with a focus on practices supporting learning, inclusion and wellbeing. It comprises a collective case study of four Australian schools that were selected based on their reputation for impactful support of students and teachers during the emergency remote teaching period. Methods included interviews and focus groups with school leaders, teachers and students. The schools had widely differing contexts, technology access and student needs. Despite these varied contexts, the findings provided important insights into common practices supporting effective remote teaching. Emerging principles of effective practice illuminate ways forward to mitigate the significant risks accompanying emergency remote teaching, and guide practices in a variety of school contexts.

## Introduction

Recent years have seen schools coping with a major disruption caused by the COVID-19 pandemic, with schools in Australia periodically required to transition from face-to-face teaching to remote teaching. This meant that school students, except children of essential workers, were required to learn from home for extended periods of time. Most teachers were expected to work from home, though a few were needed on school campuses to care for the children who needed to be on the school campus. There was minimal time for teachers to prepare for these remote teaching scenarios, hence they are referred to as ‘emergency remote teaching periods’.

This collective case study of four Australian schools investigates ways that they responded to the initial 2020 emergency remote teaching period (taking place during April to June, 2020, in the second term of the year). The schools were selected for their exemplary reputation for managing the challenges of this teaching period. While they had widely differing contexts, access and student needs, schools also shared a number of common practices. This study interrogates common pedagogies and approaches supporting student learning and wellbeing, and identifies a range of strategies shared by the schools. It also focuses on teacher wellbeing and support, an often neglected part of the literature on emergency remote teaching. The research questions are as follows:

1. What pedagogical practices have teachers used to support students’ learning during the pandemic emergency remote teaching period in 2020?
2. What practices were adopted to support students’ wellbeing and inclusion?
3. How were teachers supported during this time?

## Background

Emergency remote teaching is defined as “a temporary shift of instructional delivery to an alternate delivery mode due to crisis circumstances” (Hodges et al., 2020, para. 13) and, as such, the aim is “to provide temporary access to instruction and instructional supports in a manner that is quick to set up and is reliably available during an emergency or crisis” (Hodges et al., 2020, para. 13). According to research commissioned by the Australian Government (Armour et al., 2020; Brown et al., 2020; Masters et al., 2020), prolonged remote teaching arrangements risk poorer educational outcomes for almost half of Australian school students. At particular risk are students from low socioeconomic backgrounds, those with English as a second language, those with special learning needs, and those in rural and remote areas. There is an emerging body of literature focussed on effective practices to mitigate these risks, based around themes of school leadership, parental support, remote teaching and wellbeing.

Leadership in times of crisis is about dealing with the immediate present and minimising harm to the school community (Smith & Riley, 2012). School leaders and teachers, who know their communities and students, are best placed to develop appropriate responses to emergency remote teaching (Cowden et al., 2020). A group of Australian school leaders, interviewed about responses to the COVID-19 pandemic, reported that their main focus was on the wellbeing of their communities and maintaining open and honest communication (Longmuir, 2021). A similar study of New Zealand principals’ response to the pandemic identified the following effective leadership practices: demonstrating empathy and prioritising wellbeing; communicating frequently and effectively using a range of media; leading collaboratively and taking on a community leadership role (Thornton, 2021).

A common theme in emerging studies is the excessive burden experienced by parents required to balance domestic and employment duties with the added duties related to their children’s remote education (Armour et al., 2020). During the period of emergency remote teaching, parental involvement in their child’s education was required to be more direct, hands-on and time-intensive at a time when many parents may have been under considerable stress. Two Australian studies discussed ways of supporting parents and carers. In the first, schools were able to support families by being sensitive to particular circumstances and contexts (Armour et al., 2020). A second study showed how schools supported parents by providing resources and strategies to support learning at home (Masters et al., 2020). In the USA, experiences of remote lockdown had greater impact due to longer periods of isolation than experienced in NSW, Australia. Nevertheless, studies on US lockdowns provide valuable insights for the Australian experience. Garbe et al. (2020) investigated parents’ experiences of remote teaching during the pandemic in the US. Based on the results, the researchers recommended that educating parents about remote learning, its tools, key pedagogical concepts, and teacher–student–parent communication options, were essential in any future remote teaching period. Another USA study (Wagner, 2022) also emphasised the importance of providing parent training to support their children during remote teaching.

### Remote teaching

The pandemic has forced schools to turn to various digital solutions in response to the imperative for emergency remote teaching (Eradze et al., 2021), with most schools adopting various blended learning structures to address challenges. There are many definitions of the term ‘blended learning’ (Hrastinski, 2019). It is most commonly described as combining online and face-to-face instruction (Graham, 2006), or a mix of synchronous and asynchronous mechanisms (Dabrowski et al., 2020). Approaches where students interact over the Internet while studying from home during school closures may be regarded as blended learning (Brown et al., 2020; Dabrowski et al., 2020), as they typically involve synchronous and asynchronous strategies. Design factors that were found to be important for successful blended learning during remote teaching included provision of engaging content, opportunities for interaction with teachers and peers, and support for learners (Armour et al., 2020).

Ewing and Cooper (2021) conducted interviews with Australian students, teachers and parents to examine their experiences during the remote teaching period. They found that, although engaging students was a top priority for teachers, the majority of students felt less engaged with both their teachers and peers. Ewing and Cooper (2021) considered that, while the pandemic expedited technology adoption in schools, the underlying pedagogy and learning design proved to be a challenge for educators. A study of senior high school students in New Zealand explored how technology-mediated pedagogies (or digital pedagogies) influenced their experience of learning at home during the COVID-19 pandemic (Yates et al., 2021). The students valued both being able to make academic progress and to maintain their social and emotional wellbeing. To this end it was important that teachers provided supportive digital pedagogies that encouraged motivation and collaboration while providing flexibility and authentic learning activities. Trust and Whalen (2021) surveyed K-12 teachers (predominantly in the US) during the pandemic and found that teachers increased their use of digital tools, but these were mainly used for information delivery and management. They found that teachers were ill-prepared to use technology for remote teaching. Australian studies have also reported on teachers’ and students’ lack of readiness for remote teaching (see for example, Hodges et al., 2020; Masters et al., 2020).

### Student and teacher wellbeing

From the start of the pandemic, there was concern for student wellbeing, and there have been reports of increased mental health issues among students (Brown et al., 2020). Interviews with teachers and school leaders in the state of New South Wales (NSW), Australia revealed that the two key factors that impacted student wellbeing were community and parental anxiety, and adapting to new ways of learning (Gore et al., 2020).

Social isolation and mental health concerns were raised by children and young people with Kids Helpline in Australia during the initial remote teaching period (Nicolson et al., 2020). Drane et al. (2021) described the risks for students, particularly students from vulnerable backgrounds, while learning at home during the pandemic. These risks were long-term educational disengagement, anxiety and digital exclusion.

There have been numerous reports of stress and negative effects on teacher wellbeing resulting from the emergency remote teaching (Dabrowski, 2021). An Australia-wide survey (Ziebell et al., 2020) found that more than two-thirds of teachers indicated that they were exhausted by the additional workload. Gore et al. (2020) found that teachers and school leaders reported an increase in their workload, and also reported on heightened anxiety about the welfare of their students. Teachers felt frustrated with being unable to deliver quality lessons to students during the emergency remote teaching period (Gore et al., 2020). Finally, Kraft et al. (2020) reported that teachers with supportive working conditions, including dependable leadership, effective communication, targeted training, meaningful collaboration, fair expectations, and recognition of effort, were least likely to experience declines in their sense of success during lockdown.

This study adds to the literature base by providing rich insights and shared practices from four schools that had a reputation for effectively managing the emergency remote teaching period due to the COVID pandemic. This is one of the few studies emerging from this pandemic environment that examines *both* pedagogical practices and student/teacher wellbeing to inform educational stakeholders about school practices in these unusual and uncertain times.

## Study design

A multiple case design was used as it enabled a focus on common phenomena across selected schools within their natural settings (Stake, 2006). The research explored the experiences and practices of the teachers, leaders and students across four schools selected for their reputation in effectively managing the remote teaching period. The schools’ contexts were diverse, meeting an important criterion for a multi-case study (Stake, 2006). All data were aggregated to identify common themes across the four sites.

This study adopted an interpretive research lens, with the aim to gain an in-depth picture of the authentic experiences of teachers, leaders and students in relation to the 2020 emergency remote teaching period. From this interpretive research perspective, the study aimed to foreground participants’ human experiences and social contexts. The research design was underpinned by the belief that knowledge construction in education is seen to occur on a storied landscape that incorporates social dimensions (Merriam, 1998).

Data were collected after the NSW 2020 emergency remote teaching period, in term 4 2020 and terms 1 and 2 in 2021, before a second major lockdown later in 2021. The delay in data collection was due to the need to reduce overload for teachers as they returned to onsite classroom teaching.

### Participants

The four schools in the collective case comprised two metropolitan and two regional independent schools in NSW, Australia. Schools were selected because of their reputation for exemplary support of student and teacher wellbeing, and high satisfaction with student learning outcomes during the 2020 emergency remote teaching period. The first school in the case, Brightwater School for Girls (pseudonyms are used for all schools) is a non-selective school in metropolitan Sydney with approximately 1300 students from Pre-Kindergarten to Year 12. Data were collected remotely during Term 4, 2020. Manlala School is a selective, coeducational school in Sydney for students from Kindergarten to Year 12 drawn primarily from a particular religious and cultural group. Data were collected onsite for this second school during Term 1, 2021. The third school, Fairmeadows Primary School, is located in regional NSW. It is a small primary school with approximately 78 students from Kindergarten to Year 5. The school has an emphasis on outdoor education. Data were collected onsite during Term 2, 2021. The final school in the case, St Theresa’s School, comprised two cohorts: St Theresa’s Alternative Learning Environment catering for students with disabilities in Years 3–12, including those who have a diagnosis of anxiety, depression, PTSD, or autism; and St Theresa’s Young Parents unit, which is an accredited special assistance unit for young teenage parents in stages 5–6. The school is located in regional NSW. Data were collected virtually for both these St Theresa’s units during Term 2, 2021. The school characteristics and participants are summarised in Table 1.

**Table 1:** School characteristics and participants

School	School characteristics	Participants
1. Brightwater School for Girls	A non-selective mid-high socioeconomic status K-12 school in Sydney	School leaders (*n* = 3); Primary teachers (*n* = 3); Secondary teachers (*n* = 4, from science and creative arts); Students: secondary (*n* = 6); primary (*n* = 5)
2. Manlala School	A selective, K-12 religious coeducational school in Sydney	School leaders (*n* = 2); Secondary teachers (*n* = 7, from HSIE, science, english and mathematics); Students: secondary (*n* = 2 × 10)
3. Fairmeadows Primary School	A small K-6 school in regional NSW with an emphasis on outdoor learning	School leader (*n* = 1); Primary teachers (*n* = 4); Students: primary (*n* = 2 × 8)
4. St Theresa’s School—two units	St Theresa’s Alternative Learning Environment—A specialist unit for students with disabilities (Years 3–12) in regional NSW St Theresa’s Young Parents—A specialist unit for teens who are young parents (stages 5 & 6) in regional NSW	School leaders (*n* = 2, one from each unit); Teachers (*n* = 4, two from each unit); Students (*n* = 2 × 5, one group per unit)

School leaders nominated selected teacher participants to cover a cross-section of cohorts and (high school) disciplines, and also chose those most likely to be most informative about management of the remote teaching period. Nominated teachers were under no obligation to participate and their input was voluntary. Students were then selected by their teachers with the aim of eliciting a diversity of views in focus groups. Teachers selected students whom they anticipated would be comfortable contributing to the focus groups. Again, participation of students was purely voluntary.

While the key role of parents was mentioned by teachers and especially school leaders in the study, parents and carers were not included as participants so as not to add to their challenging circumstances working from home while balancing home schooling requirements during the pandemic.

### Methods

Multiple sources of data were used in this research, including 30-min semi-structured interviews and focus groups with school leaders and teachers across the four schools, as shown in Table 1. School leaders were interviewed individually, except at Manlala and St Theresa’s, where they were interviewed in pairs. Teacher focus groups were conducted in pairs, and for secondary teachers, the pairs were from the same discipline. Given the demands on teachers, the structure of the pairs was largely dependent on convenience factors. Student focus groups were also conducted, each lasting 30 min. Semi-structured interview and focus group schedules were prepared with the aim of interrogating phenomena of interest in situ, and promoting rich conversations with participants, which allowed discussions to roam freely within a set framework. Extracts from the prepared schedules are available in Appendix.

Formal university ethics approval was sought and received prior to commencement of the research (UTS HREC No. ETH20-5354). Principals were contacted as per industry ethics procedures, with consent sought at a broader school level prior to consent being obtained from participants or students’ parents. All participants provided consent in a written form and participants were told they were able to withdraw at any point without having to give a reason. None of the participants chose to withdraw. Member checking of the data was done in each school after collection.

### Analysis

Two researchers collected the data from each school, and subsequently analysed the aggregated data to search for common meanings relevant to the research questions. The data were categorised, resulting in a set of aggregated instances and patterns that appeared repeatedly (Stake, 1995). Emerging themes pertained to remote teaching practices supporting learning, wellbeing and inclusion. Two other researchers then examined these new meanings, to ensure intra-researcher consensus. A final set of themes was developed by the four researchers that emerged from important common meanings across the collective case and relevant to the research questions (Stake, 1995).

## Findings

The four schools addressed the challenges of the emergency remote teaching period in a range of effective ways. The findings capture the experiences from participants in all four schools with respect to their commonly adopted pedagogical approaches, and shared strategies supporting wellbeing and inclusion. Indicative examples from the collective case that illuminate themes have been provided in each sub-section.

### Pedagogical practices

The 2020 emergency remote teaching period occurred with little time for planning or preparation. Practices involved the adoption of blended learning approaches, peer learning and explicit teaching. New practices required flexibility and collaborative problem-solving from school leaders and staff, in order to embrace these new approaches and still provide continuity for students. Teachers needed to think differently and plan around available technologies and what students had access to. Schools recognised that problems would inevitably emerge and would need to be solved quickly and collaboratively. Teachers understood that they could not merely replicate classroom practices and would have to adapt to suit an online environment. What follows are pedagogical practices that were highlighted in the data. These practices were repeatedly mentioned across the data from the four schools. Illustrative examples of these practices were selected to enrich the narrative.

#### Blended learning to support student agency

Schools significantly developed their blended learning approaches during the remote teaching period, adopting structures that were suited to their own unique circumstances. These approaches included technology-mediated synchronous and asynchronous teaching, as well as a carefully tailored mix of digital and non-digital strategies. Key to these solutions in most schools was a reduction in the volume of synchronous or live classes (e.g. using Zoom video conferences), where possible. This allowed for a stronger focus on the design of learning tasks that promoted student agency by enabling student self-regulation and choice, as illustrated in the following indicative examples.

Video-conference classes at Fairmeadows Primary School were used sparingly, usually as a segue to students’ self-paced learning activities that required use of resources from their take-home learning packs. The main technologies adopted were Sway and Zoom, accompanied by the learning packs consisting of non-digital materials in a sewn fabric bag. In this way, the school adopted a mixture of ‘tech and non-tech’ strategies as part of their blended approach. Aligned with the school’s outdoor education philosophy, teachers strove to design activities suitable for learning outside in the backyard, the park or the beach. Students would record photos of experiments and later share these over video-conference classes. This integrated approach was perceived by staff to be faithful to the school’s values: “There was positive feedback from staff. In terms of our philosophy, I think it blended really well that way” (Year 4 teacher from Fairmeadows, interview). There was an emphasis on carefully selected activities, rather than volume of learning. Parents were recognised as critical to the success of this approach, and teachers supported them with targeted instructions and other resources.

Teachers at St Theresa’s Young Parents Unit also minimised live scheduled online classes. They worked instead on building their students’ capacity to learn more independently, as students were accustomed to having considerable one-on-one support. They designed self-paced activities and requested students return work samples or photographs via their Learning Management System (LMS) or email their work as evidence of their learning. Teachers were able to adjust the task for each student’s differing circumstances with their young babies at home. Staff felt that many students benefited from a more self-controlled environment and felt “empowered that they could take control of their learning” (Teacher from St Theresa’s Young Parents Unit, interview). Because these students had to rely on themselves more while learning at home, teachers noticed that they were more willing to work things out for themselves, rather than relying on the teachers to show solutions.

Two secondary creative arts teachers at Brightwater found that the student participation levels increased with the extra autonomy granted to learners. For instance, when asked to video-record their own performances at home, they found that students were less intimidated by performing in front of a camera, and the school now continues to use video-based performances. The teachers reported that a benefit was the ability to spend more time listening to the students one-on-one, and then being able to give tailored feedback via the school Learning Management System (LMS).

#### Peer learning approaches to support engagement and social interactions

Teachers used online peer learning activities not only to support engagement, but also to optimise social interactions to keep students connected and combat isolation. Teachers at all schools were conscious of these benefits and as a result, they adopted digital practices to leverage learning conversations and group work.

For the Music Director at Brightwater, the biggest challenge related to performance and collaboration—rethinking how to enable group work and interactivity. It was difficult to synchronise the collective playing of music due to ‘online delay’, so the music teachers she supervised changed their thinking about the way students could play their instruments, and still provide interactive experiences. Re-designed lessons encouraged online interactions between students, comprising singing, playing, moving, composing and creating, increasing student confidence and participation. These revised lessons enabled a rich and collaborative music experience for the students, thus mitigating the isolating nature of the lockdown. The two secondary science teacher participants at Brightwater reported on their use of online collaborative platforms such as Padlet and Google documents, so their students could collectively brainstorm and share different ideas, and readily see others’ perspectives. The students enjoyed using these co-shared facilities that provided a more permanent way of documenting ideas and conversations.

The two secondary mathematics teachers at Manlala reported on their adoption of online peer learning strategies for homework tasks, especially to support students needing additional support. Students would post their questions online and then assist each other with solutions, with teachers contributing at strategic points. One of these teachers described an online strategy he used with a Year 12 mathematics class: “I have a homework help channel, and this is where the students will ask a question and they’ll reply to one another, and help each other”.

#### Explicit teaching exploiting new media

Teachers had less time for explicit teaching during the remote teaching period. Subsequently, teachers used new media to support succinct instructional strategies. Many teachers invested much time in creating carefully tailored instructional videos, for example, which attempted to efficiently communicate key ideas and explanations to students. Teachers found this process confronting and even intimidating at first, but developed new skills in creating and enacting these instructional digital approaches. In this way, the remote teaching period became a time when many teachers developed their digital proficiencies to support explicit teaching strategies.

Fairmeadows primary school teachers were encouraged to make succinct videos that prioritised content and targeted intended learning outcomes. The Year 1 teacher commented on her instructional video recordings: “I really liked the way I reinvented my maths lessons. Because I was only given a few moments for explicit teaching each week, I had to be very succinct” (interview). These videos were typically filmed by Fairmeadows teachers in outdoor settings, for example, using their backyard as a background, to promote an outdoor aesthetic aligned with the school’s ethos. All four teachers at Fairmeadows reported that they initially found it challenging to record themselves. For instance, one teacher said: “I’m really comfortable speaking in front of students but taking a video of myself teaching, it really takes you out of your comfort zone” (Kindergarten teacher from Fairmeadows, interview). Teachers have continued to use video-based instructional strategies post lockdown as they recognised the benefits for improving the efficiency of their explicit teaching practices.

The four teacher participants from St Theresa’s reported on their creation and sharing of videos to assist students through explicit teaching practices and scaffolded instructions. They also ensured their videos were succinct, well-structured and student-friendly, and sometimes shared them in a daily blog within their school LMS. Using a flipped learning approach, they would instruct the students to watch these videos, or click on a hyperlinked image, prior to classes. Visual cues were used as a supplement to verbal and written instructions and explanations on the LMS, as an additional support for students with autism.

### Practices supporting students’ wellbeing

A fundamental commitment to students’ wellbeing underpinned decisions relating to all four schools’ adopted practices during the remote teaching period. This care was often extended to students’ families. A comment from the Fairmeadows principal was typical of the school leaders’ dedication to their students’ welfare: “It was about ensuring their physical and emotional needs were met before we focused on their academic needs” (School principal from Fairmeadows, interview). The four schools’ commitment to student wellbeing was manifested in numerous ways. Apart from pastoral care through frequent personal and online check-ins with students, family support and opportunities for online social interaction with peers were provided. Collectively, these activities enabled the schools to maintain the community connectedness, while also ensuring that students and families were quickly identified for additional support where needed.

#### Peer interactions

Supporting students’ social interactions with peers through online technologies was seen as a critical way to keep them connected and to combat isolation. At Brightwater School, peer interaction was encouraged each day through online pastoral care groups, while at Manlala, teachers encouraged students to communicate and collaborate online regarding homework and other class topics. Similarly at Fairmeadows, teachers emphasised opportunities for students to connect and talk with each other, both for their learning but also to combat isolation: “You need to allow the students to talk to each other. … If you can facilitate group work where they Zoom each other and then share ideas, that’s actually really valuable and much better for their wellbeing” (Year 1 teacher from Fairmeadows, interview). One of the students at St Theresa’s Young Parents Unit reflected on combating isolation: “…being able to keep in touch with people, and getting your mental health on track, … just keeping in contact with your teachers and friends, would be the biggest part of my experience” (Student from St Theresa’s Young Parents, focus group).

#### Support of families

As part of supporting students’ wellbeing, extra care was extended to students’ families in all schools. Regular communication with parents was critical. The previously discussed blended learning approaches gave students flexibility and autonomy with the timing and pacing of their learning, consequently helping families to manage competing home interests, supporting efficiency and wellbeing. While a range of approaches was adopted across schools, the importance of connecting with families was a consistent theme. This enabled not only a sense of connection and necessary communication, but it also provided schools with information to aid in supporting families where needed. All schools reported positive engagement by families and a gratitude for this increased vigilance.

Schools considered regular communication with families as important for supporting wellbeing. Newsletters and videos were published frequently and included details of services available to families, advice from school counselling staff and online learning advice: “The main thing was to let everybody know that we are here to support them if they need it, to reach out” (School leader from Manlala, focus group). The principal at Fairmeadows School, for example, made a daily video for families. The school surveyed parents to ask how they were coping, and reached out to those who requested assistance.

Another goal was to support parents at home who were trying to help facilitate their children’s learning, sometimes under challenging circumstances: “We were on the phone for hours, just reassuring parents, saying it’s okay, you don’t have to be perfect” (School leader from Manlala, focus group).

The flexible approach to learning used at the schools was helpful to parents’ competing needs at home, which in turn, supported families’ wellbeing. The asynchronous nature of many tasks gave children flexibility with their learning, helping parents and siblings to manage the inevitable multi-tasking challenges and sharing of digital devices experienced by families in lockdown.

### Strategies supporting student inclusion

Educators developed practices that catered for the needs and circumstances of all students, including those with additional needs, and those with home technology access or connectivity limitations.

#### Addressing digital inequities

School leaders were conscious of students who were disadvantaged in their learning due to limited access to the Internet and/or to devices. To address this problem, schools collected digital resources, such as teacher-made videos or downloaded copies of textbooks, to share with students on portable digital storage media. They also created take-home learning packages containing self-paced learning resources.

All seven Manlala teacher participants reported on their concern about students being left behind because of access issues, and invested extra time offering remedial support. Recorded lessons and archives were beneficial to students who had limited home access to technology—for example, due to siblings sharing devices. Some students received printed lesson notes which allowed them to complete learning at home in a way that worked best for them.

Most students at St Theresa’s Young Parents School had limited access to the Internet and computers at home, and were reliant on using their phones. The school used a range of approaches to support and communicate with the students, including use of emails, phone calls and systematic home ‘drop-offs’ of packs containing hard copies of paperwork and resources.

#### Individualised learning approaches

Teachers adopted individualised learning approaches where possible. These included use of personalised learning plans, use of online sessions in smaller groups, approaches that catered for students who needed to be on campus and tailoring of activities to students’ learning needs.

A priority for both St Theresa’s units was flexibility, and personally tailored approaches that were adapted to students’ special needs. This priority aligned with their school values and underpinned everyday practices in normal times: “Fundamentally, the fact that we are individualised, we are flexible, we adjust to the students that we have – this didn’t change” (School leader from St Theresa’s, focus group).

Teachers at St Theresa’s Young Parents Unit created individual learning plans and solutions based on their students’ needs: “We would see a student with a problem, and then ask, ‘how are we going to adjust to make the curriculum accessible to them’?” (Teacher from St Theresa’s, interview).

Teachers at Brightwater adjusted learning contracts to suit students’ requirements. The learning support teachers would look through the teachers’ planned contracts, and make suggestions for scaffolding activities according to students’ specific needs. There were some students whose specific needs were better suited to learning on the school campus in a more structured environment. The school contacted their parents to let them know that the option was available if they wanted their child to work on campus.

### Practices supporting teachers

School leaders recognised the potential for increased staff stress emerging from the pivot to remote teaching, and from the isolation experienced during the lockdown period. As well as ensuring effective technical support was available, they created a caring environment for teachers through enhanced staff collaboration and workload management.

#### Staff collegiality and collaboration

Staff collegiality was a driving factor in each school’s successful management of the remote teaching period, providing peer support and enhancing staff wellbeing. All schools reported on strong staff collaboration and a collective problem-solving approach that supported teachers’ development of new teaching practices, assisting with their skill development and easing anxiety. Sharing of ideas and resources was also crucial.

School leaders asked their staff to meet frequently to maintain collegiality, combat isolation and check-in with each other. At Fairmeadows, school leaders held frequent staff online meetings, beginning with a check-in to gauge how teachers were feeling. When developing digital resources, Fairmeadows teachers were encouraged to seek feedback from other staff members, and to give each other feedback about what was working. This helped bring consistency across the school and meant that teachers did not feel alone. “It was fortunate to be working as a team. Because we worked together well, that was really supportive … to share ideas and resources. It really helps when people are circulating what they’ve used” (Kindergarten teacher from Fairmeadows, interview).

All seven teacher participants at Brightwater attributed their accomplishments during the remote teaching period to working collaboratively. Like the Fairmeadows staff, the Brightwater teachers regularly met online with colleagues to support each other. They developed a renewed appreciation of the varied skills within their disciplinary teams, and the importance of teacher teamwork. These Brightwater teachers participated in their discipline-based groups with a focus on their specific expertise (e.g. video-making and other media production skills, design of online resources), thus supporting others in their team. They enjoyed considerable autonomy in these teams, and had the freedom to problem-solve within broad guidelines written by school leaders.

#### Workload management

Mindful of the importance of teachers’ wellbeing, the leaders of all four schools recognised the need to help staff manage their workload, and to set boundaries for both teachers and parents regarding workload expectations.

There was a clear endorsement of staff managing a healthy work–life balance. Because teachers were monitoring and responding to students’ online requests for assistance before and after school, they initially found it difficult to ‘switch off’ from work at the end of the day. For these reasons, the leadership teams reminded teachers to create boundaries between their work and home lives. For example at Manlala, leaders developed guidelines to advise parents of the need to set realistic expectations for teacher email replies and student learning time at home. “We advised that teachers also have families at home, so don’t expect an answer within 48 h. We had to develop guidelines for the teachers and for the families to say, ‘switch off, you don’t have to be learning all the time’.” (School leader from Manlala, focus group).

The leaders provided flexibility, rostering staff on or off school campus depending on their personal circumstances. A challenge for many teachers was not just managing their classes, but also facilitating the learning of their own children in lockdown with them. Providing flexibility helped many staff members to manage this challenge of being a parent and a teacher. At Fairmeadows School, one teacher remarked: “I did appreciate the fact that the principal put so much priority onto our wellbeing during that time” (Year 4 teacher from Fairmeadows, interview).

## Discussion

A strength of this study is the inclusion of diverse schools in the collective case study. Despite, or perhaps because of, this diversity, some clear themes arose, providing valuable insights about the remote teaching period. The findings unpack and illuminate how these schools met the shared challenges of, and developed common solutions for, teaching in the emergency remote period, providing new understandings across different contexts.

All four schools in this study emphasised that their own distinctive school ethos was key to their nuanced decision-making during remote teaching. In every school, leaders stressed that their school’s values and mission informed whole-school decisions and strategies to support teaching, learning and wellbeing during this period. For example, Fairmeadows Primary School emphasised the school’s values of ‘connect, protect, and respect’, and their mission to help children ‘learn and shine’ through authentic outdoor learning experiences. The principal and staff aimed to be faithful to these values during the remote teaching period, and used them to inform strategies supporting teaching, learning and wellbeing. So a priority for leaders and teachers during the remote teaching period was to adopt practices that provided student experiences that were aligned with their schools’ values.

Looking across the four schools, it is clear that teacher collegiality, flexibility and collaborative problem-solving among staff and school leaders were key to adopting new practices to support learning and wellbeing. Blended learning structures were developed to emphasise asynchronous activities leveraging student agency and peer learning; and explicit teaching was optimised through teacher-designed new media solutions. Student wellbeing and inclusion were a priority, and strategies were developed to support students and their families, such as frequent check-ins, increased social interactions and regular, targeted communications. Parents were well-supported, and they appreciated the flexible teaching approaches promoted by schools that allowed them to mitigate home-based challenges, such as access to devices and management of their own remote work commitments. Teacher wellbeing was also emphasised through the offering of support by the school executive, their collaboration with colleagues and the management of their workload.

The schools in the case addressed significant challenges arising from the emergency remote teaching period. Many of these problems are well documented in the literature, including: excessive burden on parents (Armour et al., 2020); additional workload of teachers (Gore et al., 2020; Ziebell et al., 2020); feeling under-prepared for pivoting to remote teaching (Trust and Whalen 2021); heightened teacher concerns about student welfare (Gore et al, 2020); finding and adjusting to suitable digital pedagogies (Ewing and Cooper 2021); student engagement and wellbeing (Bond, 2020; Ewing and Cooper 2021); access to technology (Bond, 2020), social isolation and adapting to new ways of learning (Gore et al., 2020). However, most of these studies collected data through surveys, and where there were case studies, they tended to be restricted in context or focus. This study offers a broad and rich analysis of how schools managed remote teaching.

Many solutions reported in this study also align with the literature, reiterating the importance of these strategies, and also confirming the impactful nature of our selected schools. These solutions include links to school leadership, such as open and frequent communication (Longmuir, 2021); strong school–family relationships and support of families (Armour et al., 2020); and prioritisation of wellbeing (Thornton, 2021). Solutions associated with teacher support are also confirmed in our study, including provision of supportive and flexible working conditions, experience of dependable leadership and staff collaboration (Kraft et al., 2020), and implementation of digital pedagogies emphasising student collaboration (Yates et al., 2021), interactions with peers (Armour et al., 2020), flexibility (Yates et al., 2021) and asynchronous strategies (Bond, 2020). Some of these cited studies were government reports, generated rapidly to respond to an urgent situation. This study supplements them with a carefully constructed, robust multiple case design. It also contributes to the literature through its focus on *both* student and teacher wellbeing throughout the remote teaching period. While many studies highlight the workload challenges for teachers, or the welfare of students during this period, few studies consider the wellbeing of all.

### Principles of effective practice

Three broad principles for effective practice emerged from this study.

Firstly, school leaders and teachers should respond to future periods of remote learning, for example due to COVID, floods or bushfires, by identifying the specific needs of their students and the wider school community, and developing associated solutions underpinned by their school values. In our study, teachers and executive in all schools ensured that the needs of their students in very diverse contexts were paramount in their planning for and management of the remote teaching period. Individualised learning programs and alternative digital access options were two examples of the attention to specific needs.

Secondly, school leaders and teachers need to find and practise a blended learning structure tailored to the needs of the school community. Teachers need to locate and refine an optimal balance of synchronous and asynchronous, digital and non-digital strategies, and rehearse these approaches with their students, including the use of supporting technologies. The findings suggest that effective blended learning solutions emphasise asynchronous activities that leverage self-paced, collaborative learning. Therefore, students need to become more comfortable and familiar with the extra independence that accompanies this type of learning. Similarly, teachers need a chance to develop and discuss new task designs that aim to mediate effective student learning across the varied physical and virtual learning spaces, both teacher-imposed and learner-controlled environments, encountered during remote teaching. Teacher participants in our study acknowledged the importance of this type of professional learning tailored to their needs. Hence, more staff professional learning opportunities are needed to improve current practices and prepare for these new approaches.

Finally, families need to be an integral part of schools’ planning and preparation for future lockdowns. Constructive dialogue with parents and carers is needed to contribute to schools’ preparation for disruptions, and prepare for a future with increased emphasis on intergenerational learning strategies (Burden et al., 2019). The schools in our study invested considerable time supporting families during the school lockdowns, in recognition of the key but unfamiliar and challenging role that many parents, grandparents and carers were adopting as facilitators of children’s learning.

### Future research directions

There is an imperative to examine how teachers refined their practices between initial and subsequent school lockdowns. Researchers could expand on our findings by further interrogating teachers’ changed practices after the initial remote teaching period. Further studies could expand our research on at-risk students, and those schools limited in their technology access.

It is also important to extrapolate the findings from studies such as this one to guide future schooling. To advance this agenda, we have used a futures methodology that uses baseline data from this study to generate four alternative scenarios for school education in a post-pandemic world (Kearney et al., 2022), with a particular focus on learner agency and digital technology use.

## Conclusion

The 2020 emergency remote teaching period gave schools an imperative to manage remote teaching, with a renewed emphasis on inclusion and wellbeing. Driven by their unique school ethos, the schools in this collective case study generated practices tailored to their own school community needs, supporting their students’ engagement across a range of learning environments. The study provides rich insights for schools if faced with a future need for remote teaching due to disruptions such as fire, floods or new viral variants. Findings highlight effective pedagogical approaches in times of crisis, and strategies for supporting student and teacher wellbeing, that will help illuminate ways forward in these uncertain times.

## Appendix

### Semi-structured interview schedule with leaders: sample items

What were the goals during the period of emergency remote teaching in early 2020?

What were the distinctive practices that gave this school a reputation for exemplary management of remote emergency teaching?

How did your provide support for teachers’ remote practices during the period?

What school-wide approaches promoted student inclusion and/or wellbeing?

What school-wide approaches promoted teacher wellbeing?

What are you most proud of? Would you do anything different next time? Any advice for others?

### Focus group schedule with teachers: sample items

Consider the approaches you adopted in your teaching that were effective, or proud of, during the period of emergency remote teaching in early 2020—please describe 1 activity. What made it worthwhile for students’ learning?

How did you prepare/plan for teaching in the emergency remote teaching period? What support was available and how useful was it?

How did you cater for the following groups in the emergency remote teaching period?

- Students with limited access to devices or Internet;
- Students with inclusion challenges in the emergency remote teaching period? E.g. students with disabilities.

What strategies did you use to:

- Interact with and support students in the emergency remote teaching period?
- Support your students’ wellbeing in the emergency remote teaching period?

What could be done better next time? Any advice for other teachers in a similar emergency remote teaching situation (school lockdown scenario).

### Focus groups with students: sample items

*Think about your experiences during the period of emergency remote teaching in early 2020 …*

Describe an activity or lesson that was interesting or different from your usual lessons at school? Why was it interesting? What did you learn?

How did you find doing assessment tasks at home? How did you complete these assessment tasks?

How did you interact with your friends and teachers during the lockdown?

What were the hardest parts of the lockdown? [*How did you manage that situation …*]

What advice would you give other students and teachers for next time?
